# Body mass index–stratified inflammatory predictors of insulin resistance in non-obese adults: a retrospective study

**DOI:** 10.7717/peerj.21769

**Published:** 2026-10-02

**Authors:** Aycan Acet, Turkan Pasali Kilit, Sertas Erarslan, Cumali Yalçın, Can Özlü, Çağla Özdemir

**Affiliations:** 1 Faculty of Medicine, Departmant of Internal Medicine, Kütahya Health Sciences University, Kütahya, Turkey; 2 Faculty of Medicine, Departmant of Internal Medicine, Division of Hematology, Kütahya Health Sciences University, Kütahya, Turkey; 3 Faculty of Medicine, Departmant of Family Medicine, Kütahya Health Sciences University, Kütahya, Turkey

**Keywords:** Insulin resistance, Inflammation, White blood cell count, CRP/HDL ratio, Non-obese adults, Body mass index

## Abstract

**Background:**

Insulin resistance (IR) is a key mechanism underlying type 2 diabetes mellitus and cardiometabolic disease. Although obesity-related inflammation is a major contributor to IR, increasing evidence indicates that IR may also develop in non-obese individuals. However, readily available inflammatory markers applicable to this population remain limited. We evaluated the association between inflammatory markers and insulin resistance in non-obese adults and assessed their predictive performance across body mass index (BMI) strata.

**Methods:**

This retrospective cross-sectional study included 149 non-obese adults (BMI < 30 kg/m^2^) evaluated in an internal medicine outpatient clinic between January and December 2025. Insulin resistance was defined as a homeostasis model assessment of insulin resistance (HOMA-IR) ≥ 2.5. Inflammatory markers and composite indices, including the C-reactive protein to high-density lipoprotein cholesterol (CRP/HDL) ratio, were calculated. Multivariable logistic regression, BMI-stratified analyses, and receiver operating characteristic (ROC) curve analyses were performed.

**Results:**

Insulin resistance was identified in 54 participants (36.2%). Individuals with IR had higher white blood cell (WBC) counts, CRP levels, and CRP/HDL ratios (all *p* < 0.05). In multivariable analysis, WBC count was independently associated with IR (OR = 1.407, 95% CI [1.090–1.815]; *p* = 0.009). BMI-stratified analyses showed that WBC count independently predicted IR in normal-weight individuals (OR = 1.789, 95% CI [1.218–2.627]; *p* = 0.003), whereas in overweight participants (BMI 25–30 kg/m^2^), the CRP/HDL ratio was independently associated with IR (OR = 7.793, 95% CI [1.056–57.499]; *p* = 0.044). ROC analyses demonstrated higher discriminative performance of WBC count in normal-weight individuals (AUC = 0.720) and greater predictive ability of the CRP/HDL ratio in overweight individuals (AUC = 0.695).

**Conclusions:**

Low-grade systemic inflammation is independently associated with insulin resistance in non-obese adults, with distinct inflammatory predictors across BMI categories. WBC count may reflect early immune activation in normal-weight individuals, whereas the CRP/HDL ratio appears more informative among overweight subjects.

## Introduction

Type 2 diabetes mellitus (T2DM) is a primary global health concern because of its strong link to morbidity and mortality (Park et al., 2021). Central to the pathophysiology of T2DM is insulin resistance (IR), defined as the reduced responsiveness of insulin-target tissues to normal insulin levels (Defronzo, 2009; Lee, Park & Choi, 2022). IR is closely linked to obesity, which promotes a chronic, low-grade inflammatory state known as metabolic inflammation (Amin et al., 2019; Gomez-Casado et al., 2025; Güven, 2024; Lee, Park & Choi, 2022; Xue et al., 2025). This ongoing inflammation disrupts insulin signalling pathways, highlighting the crucial need for early identification of IR, as it often precedes the development of T2DM and its complications by years (Defronzo, 2009; Lee, Park & Choi, 2022). Due to the complexity and high cost of the gold-standard method (hyperinsulinaemic euglycaemic clamp), practical surrogate indices derived from routine clinical parameters are widely adopted (Tahapary et al., 2022). Key examples include the homeostasis model assessment of insulin resistance (HOMA-IR) and the triglyceride–glucose (TyG) index, with the latter frequently demonstrating superior predictive performance for T2DM incidence compared with HOMA-IR (Park et al., 2021).

Chronic low-grade systemic inflammation contributes to insulin resistance primarily through cytokine-mediated impairment of insulin receptor signalling and disruption of glucose homeostasis in adipose tissue, liver, and skeletal muscle (Amin et al., 2019). In this context, a number of routinely available peripheral blood inflammatory markers have been investigated as accessible surrogate indicators of metabolic inflammation. Total leukocyte and neutrophil counts have been associated with features of the metabolic syndrome and insulin resistance in meta-analytic and population-based studies (Gomez-Casado et al., 2025), while leukocyte-derived composite indices, including the neutrophil-to-lymphocyte ratio (NLR) and the systemic immune-inflammation index (SII), have been correlated with glycaemic indices and insulin resistance in patients with or at risk of T2DM (Güven, 2024). More recently, the high-sensitivity C-reactive protein to high-density lipoprotein cholesterol (hs-CRP/HDL) ratio has been proposed as a composite inflammation–lipid marker that captures both the pro-inflammatory and the dyslipidaemic components of cardiometabolic risk and has shown non-linear associations with insulin resistance and T2DM in large national cohorts (Xue et al., 2025).

Despite the utility of these established indices (HOMA-IR and TyG index), their precise cut-off points for defining IR exhibit significant variability across diverse populations (age, sex, ethnicity) (Esteghamati et al., 2010; Lee et al., 2016). Furthermore, traditional measures of obesity, such as body mass index (BMI), are insufficient because they fail to accurately differentiate between fat, muscle, and bone mass, thus limiting their effectiveness in precisely assessing body fat (Gomez-Casado et al., 2025). This limitation is particularly pronounced because IR is not confined strictly to obese individuals; it also manifests in patients categorised as non-obese (Amin et al., 2019). Moreover, it remains unclear whether inflammatory predictors of insulin resistance differ according to subtle variations in adiposity within the non-obese range. Therefore, the present study aimed to investigate inflammatory predictors of insulin resistance in non-obese adults using routinely available laboratory parameters and to evaluate whether these associations differ according to BMI categories. We hypothesised that routinely available inflammatory markers are independently associated with insulin resistance in non-obese adults and that the dominant inflammatory predictors differ across body mass index categories within the non-obese range.

## Material and Methods

### Study population and patient selection

This retrospective cross-sectional study was conducted between January 2025 and December 2025 in the internal medicine outpatient clinics of a tertiary care hospital. Adult outpatients presenting with any complaints, particularly recent weight gain and appetite abnormalities, were screened. Those meeting the inclusion criteria and classified as non-obese according to the World Health Organization (WHO) criteria were subsequently included (WHO, 2025). According to WHO definitions, obesity was defined as a body mass index ≥ 30 kg/m^2^, overweight as 25.0–29.9 kg/m^2^, and normal weight as 18.5–24.9 kg/m^2^. The inclusion criteria were: being between 18 and 60 years of age, having a BMI < 30 kg/m^2^, and being admitted to the internal medicine outpatient clinic. The exclusion criteria were: age <18 or >60 years, BMI ≥ 30 kg/m^2^, acute infection, chronic inflammatory disease or chronic medical conditions requiring medications such as hypertension, diabetes mellitus, thyroiditis, and autoimmune diseases; pregnancy or lactation; use of anti-inflammatory or anti-obesity drugs (steroids, non-steroidal anti-inflammatory drugs, orlistat); history of malignancy or chemotherapy; C-reactive protein (CRP) ≥ 5 mg/L; and hospitalisation at the time of evaluation. Only patients with complete and reliable clinical and laboratory data were included. All participants were recruited from the local population and were of a similar ethnic background. The control group consisted of individuals without insulin resistance. Demographic and anthropometric data, including age, sex, height, weight, BMI, and waist circumference, as well as biochemical parameters, were collected from the hospital information system at the time of outpatient admission.

### Ethics approval and consent to participate

This study was approved by the Kütahya Health Sciences Medical School Institutional Ethics Committee (Decision No: 2025/13-16; Date: November 18, 2025) and was conducted in accordance with the Declaration of Helsinki. Due to the retrospective nature of the study, the requirement for written informed consent was waived by the Ethics Committee.

### Laboratory measurements

Fasting venous blood samples were obtained from all participants between 08:00 and 10:00 after an overnight fast of at least eight hours, in accordance with routine outpatient clinical practice. All biochemical measurements were performed in the central clinical laboratory of our hospital. Fasting plasma glucose, total cholesterol (TC), low-density lipoprotein cholesterol (LDL), high-density lipoprotein cholesterol (HDL), triglycerides (TG), creatinine, uric acid, alanine aminotransferase (ALT), and aspartate aminotransferase (AST) were measured by standard automated spectrophotometric enzymatic methods. Fasting insulin was measured by an electrochemiluminescence immunoassay. Glycated haemoglobin (HbA1c) was measured by high-performance liquid chromatography. C-reactive protein was measured by a standard immunoturbidimetric assay. The complete blood count, including white blood cell (WBC), neutrophil, lymphocyte, monocyte and platelet counts, and mean platelet volume (MPV), was measured on an automated haematology analyser.

### Calculation of insulin resistance and inflammatory indices

Insulin resistance was calculated using the Homeostasis Model Assessment for Insulin Resistance (HOMA-IR) according to the formula originally described by Matthews et al. (1985). The classification of insulin resistance was based on a HOMA-IR threshold of 2.5 (Yamada et al., 2011).

### HOMA-IR = (fasting glucose [mg/dL] × fasting insulin [μU/mL])/405

Inflammatory indices were computed as follows:

• **Systemic Immune-Inflammation Index (SII):** (Neutrophil count [10^3^/µL] × Platelet count [10^3^/µL])/Lymphocyte count [10^3^/µL]
• **Neutrophil-to-Lymphocyte Ratio (NLR):** Neutrophil count [10^3^/µL]/Lymphocyte count [10^3^/µL]
• **Platelet-to-Mean Platelet Volume Ratio:** Platelet count [10^3^/µL]/MPV [fL]
• **CRP/HDL Ratio:** CRP [mg/L]/HDL cholesterol [mg/dL]

### Statistical analysis

Data were summarised in tables and analysed using appropriate statistical tests with IBM SPSS Statistics Version 30.0 (IBM Corp., Armonk, NY, USA). The normality of data distribution was assessed using the Shapiro–Wilk test. Categorical variables were compared with the chi-square test, and continuous variables were analysed using either the Student’s *t*-test or the Mann–Whitney *U* test, according to data distribution. Because several inflammatory and metabolic variables exhibited right-skewed distributions, logarithmic transformation was applied when appropriate prior to regression analyses. Initially, univariate logistic regression analysis was performed to identify variables associated with insulin resistance. Multicollinearity among independent variables was evaluated using correlation matrices and variance inflation factors (VIF). To avoid collinearity and overfitting, variables reflecting similar biological pathways were not entered simultaneously into multivariable models. Accordingly, WBC count, BMI, and the CRP/HDL ratio were selected for multivariable analysis based on clinical relevance and statistical significance in univariate analyses. A stratified multivariable logistic regression analysis was conducted across BMI categories to identify independent predictors of insulin resistance. The discriminative performance of significant variables was assessed using receiver operating characteristic (ROC) curve analysis. ROC analyses were intended to evaluate risk stratification rather than diagnostic accuracy. All statistical analyses were two-tailed, and a *p*-value < 0.05 was considered statistically significant.

## Results

A total of 149 non-obese adults were included in the analysis, comprising 91 normal-weight (BMI 18–25 kg/m^2^) and 58 overweight (BMI 25–30 kg/m^2^) individuals. Baseline demographic, anthropometric, and laboratory characteristics stratified by BMI category are presented in Table 1. Overweight participants had significantly higher BMI, waist circumference, ALT, uric acid, triglyceride, LDL cholesterol, HbA1c, insulin levels, WBC count, and neutrophil count compared with normal-weight individuals (all *p* < 0.05). Conversely, HDL cholesterol levels were significantly lower in the overweight group (*p* = 0.003). No significant differences were observed between BMI groups with respect to AST, platelet count, MPV, or SII (*p* > 0.05). Among composite inflammatory indices, the CRP/HDL ratio was significantly higher in overweight individuals compared with normal-weight participants (*p* < 0.001), whereas NLR, platelet/MPV ratio, and SII did not differ significantly between BMI categories.

**Table 1:** Comparison of demographic and laboratory parameters in non-obese (normal weight and overweight) patients.

	**All patients (*n* = 149)**	**Normal Weight** **(BMI: 18–25 kg/m^2^) (*n* = 91)**	**Overweight** **(BMI: 25–30 kg/m^2^)** **(*n* = 58)**	***p*** **value**
Age (year)	31.34 ±11.78	30.62 ±9.60	32.17 ±12.57	0.675
Gender, male (%) *δ*	29.5%	25.3%	36.2%	0.076
BMI (kg/m^2^)	24.16 ±3.27	22.03 ±2.12	27.49 ±1.50	<0.001
Waist circumference (cm)	83.70 ±12.32	77.32 ±8.77	93.71 ±10.0	<0.001
IR	2.38 ±1.62	2.17 ±1.48	2.71 ±1.79	0.036
Glucose (mg/dL)^*^	91.01 ±8.89	90.13 ±9.36	92.38 ±7.97	0.133
Creatinine (mg/dL)	0.75 ±0.15	0.74 ±0.15	0.78 ±0.15	0.116
eGFR (mL/min/1.73m^2^)	112.44 ±15.14	114.80 ±14.12	100.61 ±16.10	0.128
ALT (U/L)	17.23 ±11.62	15.09 ±9.01	20.59 ±14.27	0.006
AST (U/L)	18.36 ±9.88	18.27 ±11.67	18.51 ±6.12	0.102
Uric acid (mg/dL)^*^	4.75 ±1.24	4.53 ±1.19	5.11 ±1.26	0.021
Triglyceride (mg/dL)	101.26 ±57.01	83.22 ±40.04	129.26 ±67.59	<0.001
HDL (mg/dL)	51.97 ±13.74	54.31 ±13.53	48.29 ±13.36	0.003
LDL (mg/dL)	100.62 ±33.37	94.55 ±31.98	110.05 ±33.55	0.006
HbA1c (%)	5.25 ±0.27	5.18 ±0.26	5.35 ±0.24	<0.001
Insulin (µU/mL)	11.16 ±7.41	10.41 ±7.84	12.33 ±6.59	0.008
TSH (mU/L)	2.08 ±1.17	1.96 ±0.92	2.25 ±1.48	0.409
WBC (×10^3^/μL)	7.04 ±1.61	6.68 ±1.42	7.60 ±1.73	0.003
Neutrophil count (×10^3^/μL)	3.93 ±1.19	3.68 ±0.96	4.31 ±1.40	0.011
Lymphocyte count (×10^3^/μL)^*^	2.33 ±0.65	2.22 ±0.61	2.52 ±0.68	0.007
Monocyte (×10^3^/μL)	0.53 ±0.15	0.52 ±0.13	0.55 ±0.17	0.211
Platelet (×10^3^/μL)	272.95 ±56.95	272.05 ±50.21	274.36 ±66.60	0.787
MPV (fL)^*^	10.33 ±0.84	10.33 ±0.79	10.32 ±0.92	0.962
Sedim. (mm/h)	5.07 ±3.81	4.62 ±2.93	5.77 ±4.84	0.263
C-Reactive Protein (mg/L)	1.78 ±1.22	1.59 ±1.19	2.07 ±1.22	0.002
CRP/HDL	0.03 ±0.02	0.031 ±0.025	0.047 ±0.031	<0.001
SII (×10^3^/μL)	493.22 ±238.96	479.16 ±191.76	515.29 ±298.99	0.758
Platelet/MPV	26.68 ±6.63	26.62 ±6.03	26.77 ±7.56	0.923
Neutrophil/Lymphocyte^*^	1.78 ±0.69	1.75 ±0.61	1.83 ±0.81	0.832

**Notes.**

Data are presented as mean ±SD.

Comparisons between groups were made using *Student’s t-test; *δ* chi-square test; otherwise Mann–Whitney U test.

BMI Body mass index IR Insulin resistance eGFR Estimated glomerular filtration rate HbA1c Hemoglobin A1c TSH Thyroid-stimulating hormone WBC White blood cell AST Aspartate aminotransferase ALT Alanine aminotransferase MPV Mean platelet volume SII Systemic inflammatory index HDL High-Density Lipoprotein Sedim Sedimentation CRP C-reactive protein

According to Table 2, 54 participants (36.2%) were classified as insulin resistant (IR+), while 95 participants (63.8%) were insulin sensitive (IR–). Age and sex distribution did not differ between groups (*p* > 0.05). Compared with IR(−) individuals, the IR(+) group exhibited significantly higher BMI, waist circumference, fasting glucose, HbA1c, insulin, ALT, and triglyceride levels (all *p* < 0.01). Inflammatory parameters, including WBC, neutrophil, lymphocyte, and monocyte counts, and CRP, were also significantly elevated in the IR(+) group (*p* < 0.05). Among composite inflammatory markers, only the CRP/HDL ratio was significantly higher in IR(+) individuals (*p* = 0.016), whereas NLR, platelet/MPV ratio, and SII did not show significant differences between IR groups.

**Table 2:** Comparision of the clinical and demographic characteristics of patients with and without insulin resistance.

	**All patients** **(*n* = 149)**	**Insulin resistance(+)** **(*n* = 54)**	**Insulin resistance(−)** **(*n* = 95)**	***p*** **value**
Age (year)	31.34 ±11.78	31.69 ±12.52	31.14 ±11.40	*0.920*
Gender, male (%) *δ*	29.5%	27.8%	30.5%	*0.724*
BMI (kg/m^2^)	24.16 ±3.27	25.21 ±3.25	23.56 ±3.15	*0.003*
Waist circumference (cm)	83.70 ±12.32	87.74 ±13.02	81.40 ±11.18	*0.004*
IR	2.38 ±1.62	4.00 ±1.54	1.45 ±0.68	*<0.001*
Glucose (mg/dL)^*^	91.01 ±8.89	95.89 ±7.98	88.23 ±8.19	*<0.001*
Creatinine (mg/dL)	0.75 ±0.15	0.77 ±0.16	0.75 ±0.14	*0.475*
eGFR (mL/min/1.73 m^2^)	112.44 ±15.14	110.97 ±18.18	113.25 ±13.25	*0.822*
ALT (U/L)	17.23 ±11.62	20.47 ±12.11	15.39 ±10.98	*0.002*
AST (U/L)	18.36 ±9.88	18.80 ±5.67	18.11 ±11.65	*0.105*
Uric acid (mg/dL)^*^	4.75 ±1.24	4.89 ±1.37	4.68 ±1.17	*0.414*
Triglyceride (mg/dL)	101.26 ±57.01	121.35 ±70.57	89.72 ±43.94	*0.005*
HDL (mg/dL)	51.97 ±13.74	50.44 ±15.74	52.83 ±12.47	*0.063*
LDL (mg/dL)	100.62 ±33.37	105.37 ±34.51	97.90 ±32.57	*0.446*
HbA1c (%)	5.25 ±0.27	5.32 ±0.22	5.21 ±0.28	*0.022*
Insulin (µU/mL)	11.16 ±7.41	17.31 ±9.10	7.66 ±2.41	*<0.001*
TSH (mU/L)	2.08 ±1.17	2.23 ±1.39	1.99 ±1.02	*0.284*
WBC (×10^3^/μL)^*^	7.04 ±1.61	7.70 ±1.58	6.67 ±1.51	*<0.001*
Neutrophil count (×10^3^/μL)	3.93 ±1.19	4.26 ±1.37	3.74 ±1.03	*0.034*
Lymphocyte count (×10^3^/μL)^*^	2.33 ±0.65	2.59 ±0.61	2.19 ±0.64	*<0.001*
Monocyte (×10^3^/μL)	0.53 ±0.15	0.57 ±0.16	0.51 ±0.14	*0.036*
Platelet (×10^3^/μL)	272.95 ±56.95	275.33 ±66.68	271.60 ±50.93	*0.920*
MPV (fL)^*^	10.33 ±0.84	10.33 ±0.91	10.33 ±0.81	*0.984*
Sedim. (mm/h)	5.07 ±3.81	5.15 ±3.52	5.02 ±3.99	*0.248*
C- Reactive Protein (mg/L)	1.78 ±1.22	2.06 ±1.30	1.61 ±1.15	*0.023*
Neutrophil/Lymphocyte^*^	1.78 ±0.69	1.75 ±0.80	1.80 ±0.63	*0.126*
Platelet/MPV	26.68 ±6.63	26.91 ±7.84	26.55 ±5.90	*0.939*
SII	493.22 ±238.96	496.32 ±297.13	491.46 ±200.30	*0.228*
CRP/HDL^*^	0.03 ±0.02	0.04 ±0.03	0.03 ±0.02	*0.016*

**Notes.**

Data are presented as mean ±SD.

Comparisons between groups were made using the *Student *t* test, *δ* Chi-Square Test, Mann–Whitney *U* Test.

BMI Body mass index IR Insulin resistance eGFR Estimated glomerular filtration rate HbA1c Hemoglobin A1c TSH Thyroid-stimulating hormone WBC White blood cell count AST Aspartate aminotransferase ALT Alanine aminotransferase MPV Mean platelet volume SII Systemic ınflammatory ındex HDL High-density lipoprotein CRP C-reactive protein Sedim Sedimentation Waist circum Waist circumference

In univariate logistic regression analysis (Table 3), several variables were significantly associated with insulin resistance, including BMI, fasting glucose, ALT, triglycerides, HbA1c, WBC count, neutrophil count, lymphocyte count, monocyte count, CRP, and log (CRP/HDL) (*p* < 0.05). In the multivariate logistic regression model, after adjustment for clinically relevant variables and multicollinearity, WBC count remained the only independent predictor of insulin resistance (OR = 1.407, 95% CI [1.090–1.815]; *p* = 0.009), indicating an independent association rather than a causal relationship. The model demonstrated acceptable goodness-of-fit (Hosmer–Lemeshow *p* = 0.425; Nagelkerke *R*^2^ = 18.9%).

**Table 3:** Univariate and multivariate binary logistic regression analyses of patients with insulin resistance.

**Variables**	**Univariate**			**Multivariate**		
	**OR**	**95% CI**	***p***	**OR**	**95% CI**	***p***
Age, year	1.004	0.976–1.033	*0.784*			
Gender, male	1.142	0.546–2.391	*0.724*			
BMI (kg/m^2^)	1.178	1.054–1.316	*0.004*	1.099	0.974–1.241	*0.125*
Glucose (mg/dL)	1.125	1.070–1.182	*<0.001*			
Creatinine (mg/dL)	2.728	0.316–23.541	*0.361*			
eGFR (mL/min/1.73 m^2^)	0.450	0.965–1.016	*0.450*			
ALT (U/L)	1.039	1.007–1.073	*0.017*			
AST (U/L)	1.007	0.974–1.041	*0.681*			
Uric acid (mg/dL)	1.145	0.829–1.582	*0.411*			
Triglyceride (mg/dL)	1.010	1.004–1.017	*0.002*			
HDL (mg/dL)	0.987	0.963–1.012	*0.309*			
LDL (mg/dL)	1.007	0.997–1.017	*0.193*			
T. Cholesterole (mg/dL)	1.007	0.998–1.016	*0.155*			
HbA1c (%)	5.265	1.346–20.604	*0.017*			
TSH (mU/L)	1.1188	0.888–1.588	*0.246*			
WBC (×10^3^/μL)	1.541	1.214–1.956	*<0.001*	1.407	1.090–1.815	*0.009*
Neutrophil count (×10^3^/μL)	1.454	1.080–1.956	*0.013*			
Lymphocyte count (×10^3^/μL)	2.673	1.517–4.708	*<0.001*			
Monocyte (×10^3^/μL)	2.154	1.270–11.299	*0.030*			
Platelet (×10^3^/μL)	1.001	0.995–1.007	*0.700*			
MPV (fL)	1.004	0.674–1.496	*0.983*			
C-reactive protein (mg/L)	1.343	1.022–1.766	*0.034*			
Neutrophil/Lymphocyte	0.908	0.557–1.480	*0.697*			
Platelet/MPV	1.008	0.959–1.061	*0.749*			
SII (×10^3^/μL)	1.000	0.999–1.001	*0.905*			
Log (CRP/HDL)	3.965	1.337–11.762	*0.013*	2.038	0.621–6.689	*0.240*

**Notes.**

Hosmer–Lemeshow (*χ*^2^ (8) = 8.087, *p* = 0.425), Nagelkerke R^2^ 18.9%.

BMI Body mass index eGFR Estimated glomerular filtration rate ALT Alanine aminotransferase AST Aspartate amino transferase CRP C-reactive protein HDL High density lipoprotein LDL Low density protein TSH Thyroid stimulating hormone WBC white blood cell count MPV Mean platelet volume SII Systemic inflammatory index

As shown in Table 4, BMI-stratified multivariable logistic regression analyses revealed differential predictors of insulin resistance across adiposity categories. In the normal-weight subgroup (BMI < 25 kg/m^2^), WBC count was identified as the sole independent predictor of insulin resistance (OR = 1.789, 95% CI [1.218–2.627]; *p* = 0.003). In contrast, in the overweight subgroup (BMI 25–30 kg/m^2^), WBC count was no longer statistically significant (*p* = 0.414). Instead, log (CRP/HDL) emerged as an independent predictor of insulin resistance (OR = 7.793, 95% CI [1.056–57.499]; *p* = 0.044). In line with the BMI-stratified analyses, insulin resistance was characterised by higher WBC counts in normal-weight individuals, whereas elevated CRP/HDL ratios predominated among overweight individuals, highlighting distinct inflammatory patterns across adiposity categories (Figs. 1 and 2).

**Table 4:** BMI-stratified logistic regression analysis of factors associated with insulin resistance.

**Variables**	**BMI <25 kg/m^2^ (*n* = 92)**			**BMI 25–30 kg/m^2^ (*n* = 59)**		
	**OR**	**95% CI**	***p***	**OR**	**95% CI**	***p***
Age	1.006	0.949–1.067	*0.839*	0.609	0.943–1.035	*0.988*
Gender (Male)	3.090	0.537–17.795	*0.207*	0.696	0.220–2.200	*0.537*
WBC (10^3^/µL)	1.789	1.218–2.627	*0.003*	1.162	0.811–1.665	*0.414*
Log (CRP/HDL)	0.781	0.382–1.595	*0.865*	7.793	1.056–57.499	*0.044*

**Notes.**

BMI Body mass index WBC White blood cell count Log Logarithmic transformation CRP C-reactive protein HDL High density lipoprotein

**Figure 1:**
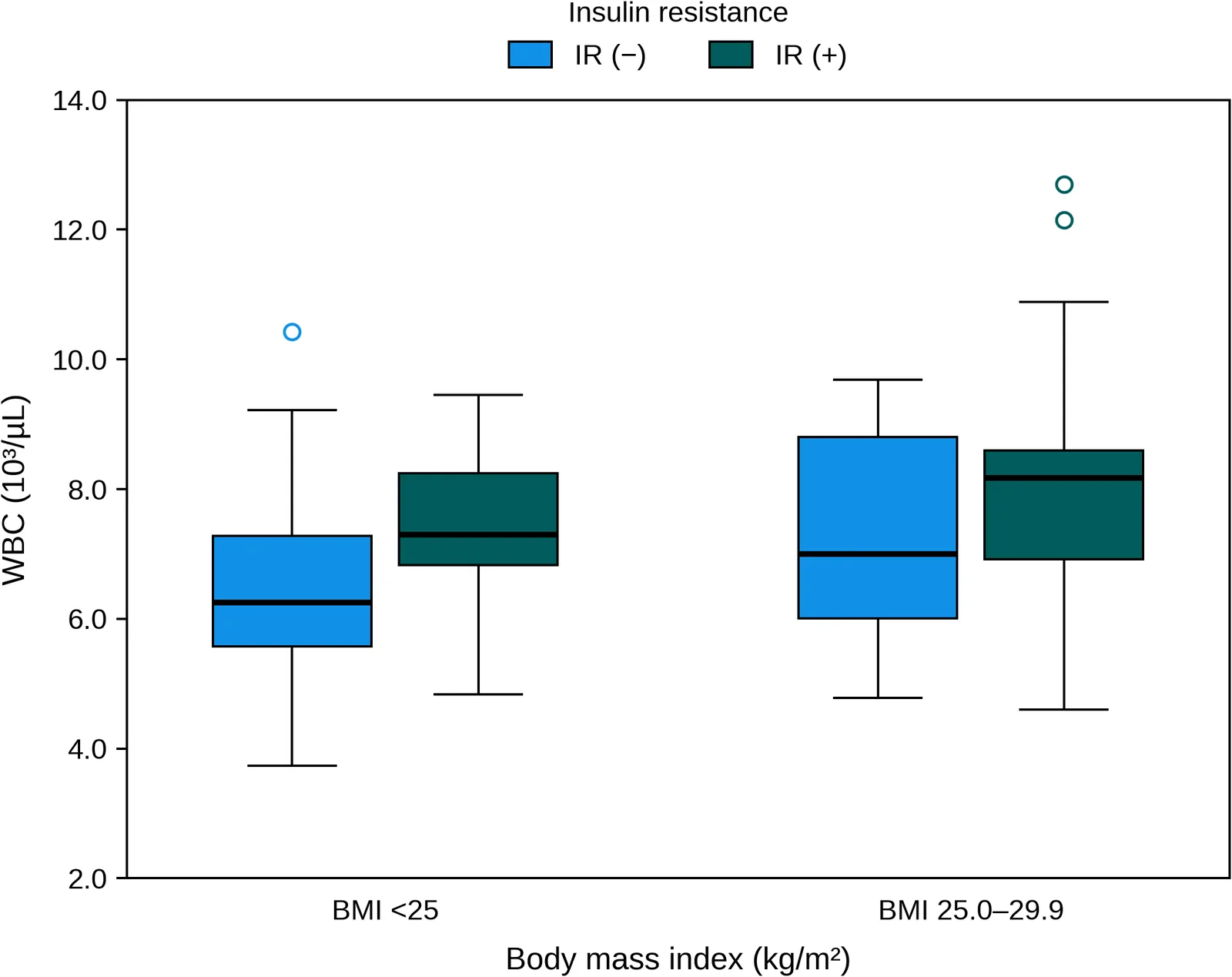
Body mass index–stratified differences in white blood cell count by insulin resistance status. Abbreviations: BMI, Body mass index; WBC, White blood cell count; IR, Insulin resistance.

**Figure 2:**
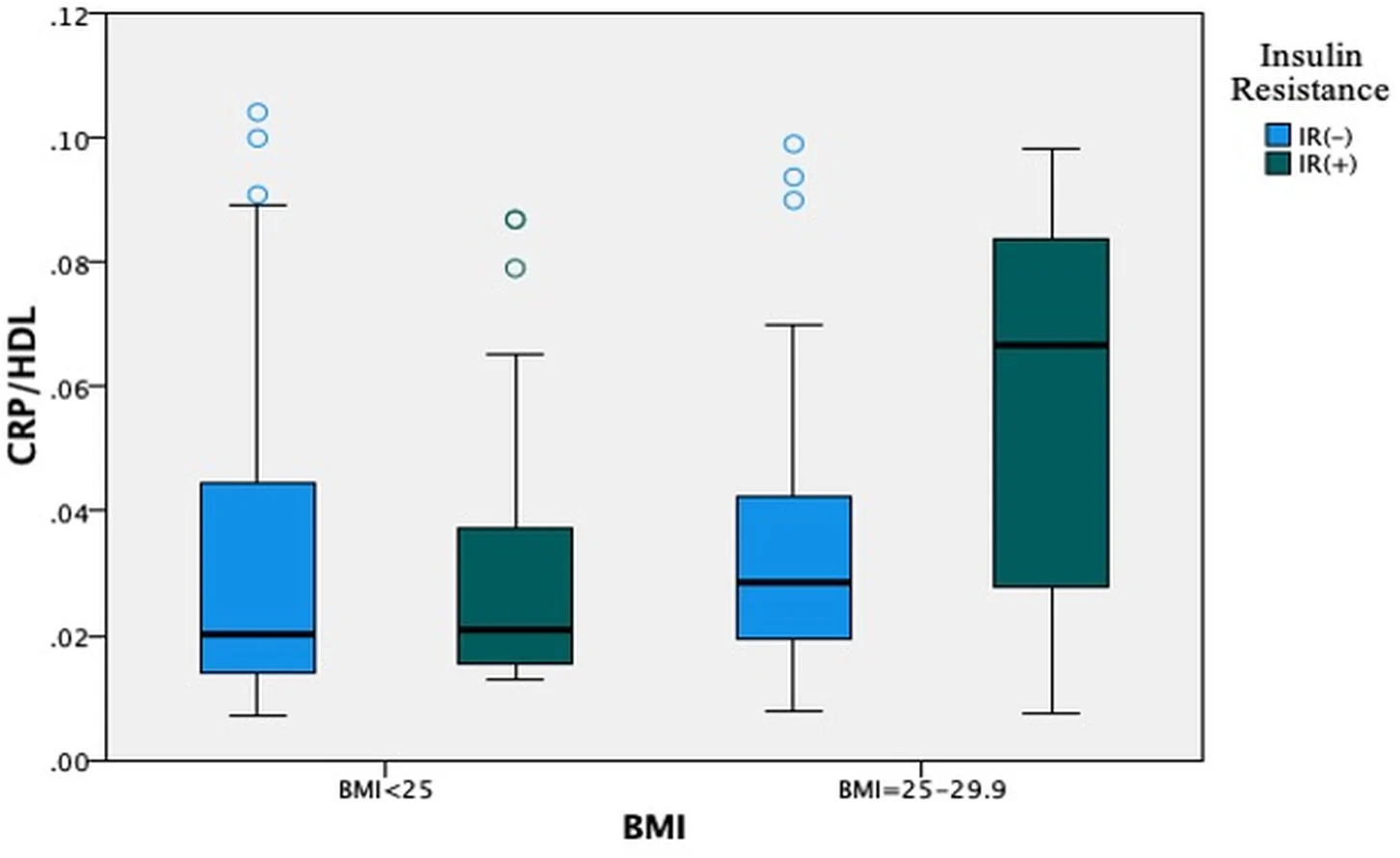
Body mass index–stratified differences in CRP/HDL ratio by insulin resistance status. Abbreviations: BMI, Body mass index; CRP, C-reactive protein; HDL, High density lipoprotein; IR, Insulin resistance.

Receiver operating characteristic curve analyses stratified by BMI category are summarised in Table 5 and illustrated in Fig. 3. Among normal-weight individuals (BMI < 25 kg/m^2^), WBC count demonstrated improved discriminative performance, with an area under the curve (AUC) of 0.720 (95% CI [0.605–0.835]; *p* < 0.001) and an optimal cut-off value of 7.11 × 10^3^/µL. In contrast, the predictive performance of the CRP/HDL ratio in this subgroup was limited (AUC = 0.547; *p* = 0.449). In the overweight subgroup (BMI 25–30 kg/m^2^), the discriminative ability of WBC count decreased (AUC = 0.590; *p* = 0.243). However, the CRP/HDL ratio exhibited significantly enhanced predictive performance, with an AUC of 0.695 (95% CI [0.555–0.836]; *p* = 0.006) and an optimal cut-off value of 0.045, reflecting higher specificity for identifying insulin resistance in this subgroup. Similarly, BMI demonstrated moderate discriminative ability for insulin resistance among overweight individuals (AUC = 0.668; *p* = 0.020), whereas its predictive value was not significant in normal-weight participants.

**Table 5:** BMI-stratified ROC curve-based evaluation of predictors of insulin resistance.

**Variable**	**Cut-off**	**Sensitivity**	**Specificity**	**AUC**	**95% CI (AUC)**	***p*** **-value**
WBC (10^3^/µL) < 25	7.11	0.74	0.73	0.720	0.605–0.835	*<0.001*
WBC (10^3^/µL) =25 − 29.9	6.55	0.85	0.46	0.590	0.439–0.740	*0.243*
BMI (kg/m^2^) < 25	22.52	0.63	0.40	0.591	0.463–0.720	*0.163*
BMI (kg/m^2^) =25 − 29.9	28.06	0.55	0.77	0.668	0.527–0.809	*0.020*
CRP/HDL < 25	0.013	0.96	0.25	0.547	0.426–0.668	*0.449*
CRP/HDL =25 − 29.9	0.045	0.59	0.80	0.695	0.555–0.836	*0.006*

**Notes.**

BMI Body mass index WBC White blood cell count AUC Area under curve ROC Receiver operating characteristic CRP C-reactive protein HDL High density lipoprotein

**Figure 3:**
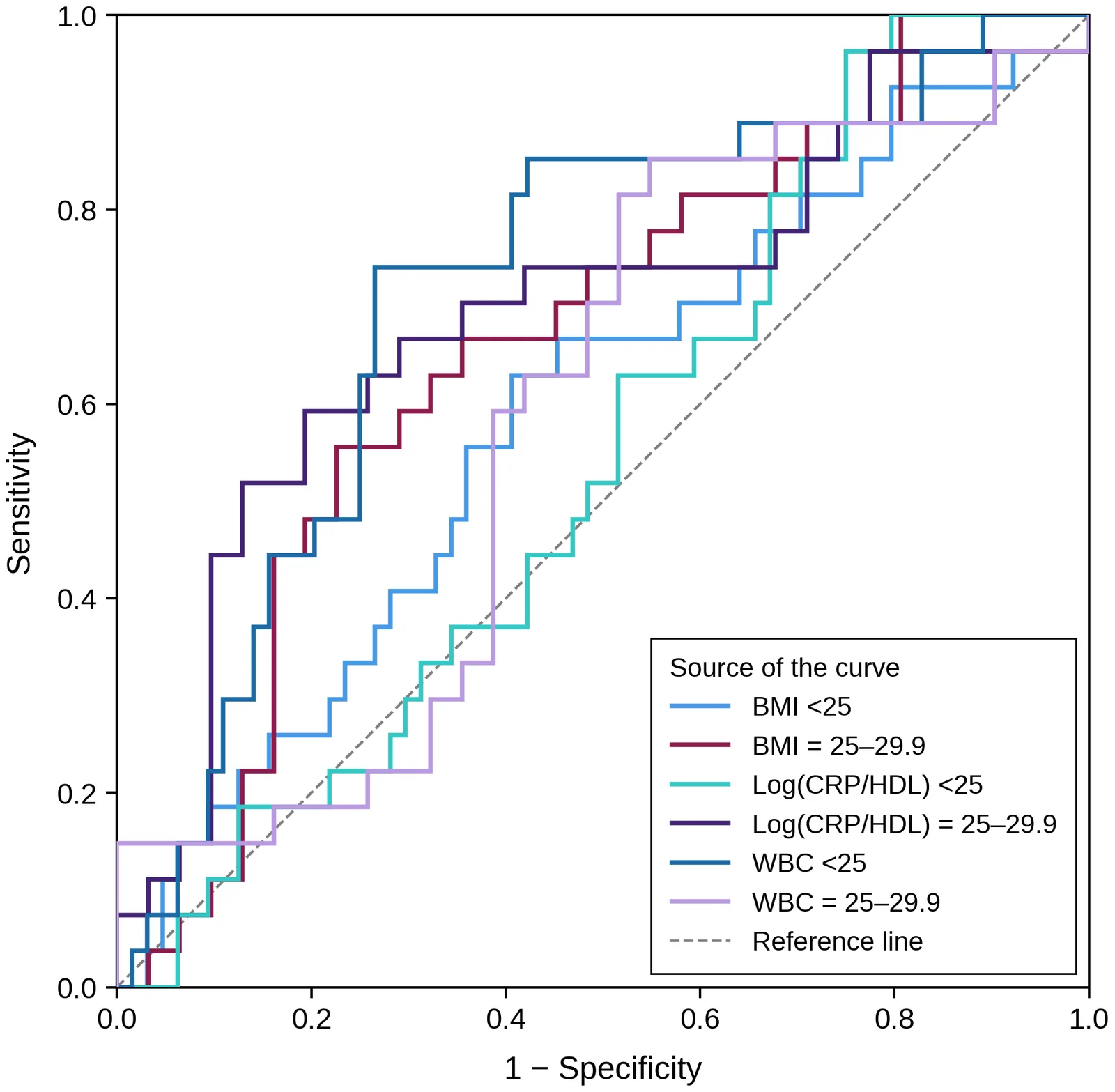
ROC curves illustrating the discriminative ability of inflammatory and anthropometric markers for insulin resistance. Abbreviations: ROC, Receiver operating characteristic; CRP, C-reactive protein; HDL, High density protein; Log, Logarithmic transformation.

## Discussion

In the present study, we demonstrated that low-grade systemic inflammation is independently associated with insulin resistance in non-obese adults and that the predictive value of inflammatory markers varies according to body mass index. The principal finding is that WBC count emerged as an independent predictor of insulin resistance in normal-weight individuals, whereas the CRP/HDL ratio was more strongly associated with insulin resistance among overweight but non-obese individuals. These results suggest the presence of distinct inflammatory phenotypes underlying insulin resistance across different adiposity strata below the obesity threshold. To the best of our knowledge, this study is among the first to investigate inflammatory predictors of insulin resistance in a young and middle-aged Turkish non-obese cohort. Although insulin resistance is increasingly recognised as a condition that may develop independently of obesity, validated surrogate markers applicable to non-obese populations remain limited. Most existing evidence originates from obese cohorts or relies on complex indices with limited applicability in routine clinical practice.

Insulin resistance and T2DM are major public health concerns driven by metabolic dysfunction and chronic, low-grade systemic inflammation (Hotamisligil, 2008). IR represents a central pathogenic mechanism linking T2DM with non-alcoholic fatty liver disease, atherosclerosis, and cardiovascular disease (Stefan, Schick & Häring, 2017). Early identification of insulin resistance is therefore essential, as intervention at this stage may prevent or delay cardiometabolic complications. Accordingly, identifying readily available biomarkers that reflect early metabolic disruption remains a clinical priority.

In non-obese individuals, insulin resistance is frequently attributed to a lipodystrophy-like phenotype characterised by limited expandability of subcutaneous adipose tissue. This promotes ectopic lipid deposition in visceral fat, liver, and skeletal muscle, leading to the accumulation of toxic lipid intermediates such as diacylglycerol and ceramides. These metabolites activate protein kinase-mediated signalling pathways, impair insulin signalling, and promote inflammation (Morino, Petersen & Shulman, 2006; Ruderman et al., 1998; Stefan, Schick & Häring, 2017). This mechanistic framework explains the development of insulin resistance in the absence of overt obesity. Our BMI-stratified findings are consistent with this framework, suggesting that early insulin resistance in leaner individuals may be more closely linked to cellular immune activation that accompanies early lipid-driven signalling, rather than to the hepatic acute-phase response that characterises more advanced adiposity.

Beyond this broad framework, several complementary pathways help to explain why white blood cell count and the CRP/HDL ratio dominate in different BMI strata. In normal-weight individuals, early ectopic lipid deposition and modest expansion of visceral adipose tissue may impair insulin signalling through intracellular accumulation of diacylglycerol and ceramides and through mitochondrial dysfunction, before the systemic hepatic acute-phase response becomes prominent (Morino, Petersen & Shulman, 2006; Stefan, Schick & Häring, 2017). At this stage, the inflammatory signal is predominantly cellular: activated innate immune cells infiltrate dysfunctional adipose tissue and the liver, and peripheral leukocyte mobilisation is reflected in a higher circulating WBC count even when CRP remains low. As adipose tissue mass expands further within the overweight range, adipocyte hypertrophy and hypoxia amplify the release of pro-inflammatory cytokines such as interleukin-6 and tumour necrosis factor-*α*, which drive hepatic synthesis of CRP and concurrently attenuate HDL-mediated anti-inflammatory capacity (Amin et al., 2019; Hotamisligil, 2008). Consequently, the CRP/HDL ratio, a composite marker that integrates both limbs of this process, becomes a more sensitive signal of insulin resistance in overweight individuals. This interpretation is internally consistent with the progressive rise in CRP and CRP/HDL across BMI strata observed in our cohort (Table 1), and it provides a biologically plausible substrate for the quantitatively distinct BMI-specific associations that we observed in the multivariable and ROC analyses.

Our findings indicate that incremental increases in BMI within the non-obese range are accompanied by elevations in inflammatory markers, including WBC count, neutrophil count, CRP, and the CRP/HDL ratio, suggesting the presence of low-grade systemic inflammation prior to the development of obesity. The progressive increase in the CRP/HDL ratio with rising BMI in our cohort underscores a shift toward a pro-inflammatory and pro-atherogenic metabolic profile. This composite marker integrates systemic inflammation and impaired lipid-mediated anti-inflammatory protection, capturing key dimensions of metabolic risk. Population-based studies have consistently demonstrated associations between inflammation-to-HDL indices most commonly formulated as the hs-CRP/HDL ratio and both insulin resistance and T2DM risk (Sun et al., 2025; Xue et al., 2025). Furthermore, the CRP/HDL-C ratio has been associated with major adverse cardiovascular events, diabetic retinopathy, and metabolic syndrome, supporting its role as an integrative metabolic risk marker (Djesevic et al., 2023; Freeman et al., 2002; Jiang & Yu, 2025; Pocovi-Gerardino et al., 2020; Sun et al., 2025). In line with these observations, BMI-stratified ROC analyses in the present study demonstrated enhanced predictive performance of the CRP/HDL ratio specifically among overweight individuals.

Among the evaluated parameters, WBC count showed the strongest predictive performance for insulin resistance, whereas the CRP/HDL ratio demonstrated lower sensitivity but higher specificity, suggesting a complementary role in risk stratification. Elevated WBC levels reflect early systemic immune activation, which is increasingly recognised as a hallmark of metabolic distress and cardiovascular risk (Chen et al., 2010; Gungoren et al., 2015; Nakanishi et al., 2002). Notably, BMI-stratified analyses revealed that the discriminative ability of WBC count was more pronounced in normal-weight individuals, whereas the predictive contribution of the CRP/HDL ratio became more evident with increasing adiposity.

The dominant inflammatory marker associated with insulin resistance varied according to adiposity status. This finding suggests a potential transition from predominantly cellular inflammatory activation in leaner individuals toward a combined inflammatory and lipid dysregulation phenotype as BMI approaches the obesity threshold. The consistency of this pattern across both multivariable regression models and ROC curve analyses strengthens the biological plausibility of this transition.

In contrast, no significant associations were observed between insulin resistance and complex ratio-based indices such as NLR, SII, or platelet/MPV ratios. These markers are typically elevated in conditions characterised by high-grade systemic inflammation and advanced disease states (Balta et al., 2013; Deng et al., 2021; Fabbrini et al., 2014; Islam, Satici & Eroglu, 2024; Shah et al., 2012; Yu et al., 2017; Zhao et al., 2024). The absence of significant associations between insulin resistance and composite indices such as NLR, SII, or platelet/MPV ratio may reflect the low-grade and subclinical nature of inflammation in non-obese individuals. In this early metabolic stage, absolute leukocyte counts may be more sensitive than ratio-based indices that are typically elevated in advanced inflammatory states. Supporting this interpretation, meta-analyses in obese populations have shown that absolute leukocyte parameters are more stable indicators of chronic metabolic inflammation (Gomez-Casado et al., 2025).

From a clinical perspective, our findings suggest a practical, BMI-tailored interpretive framework for the early detection of insulin resistance in non-obese adults using only routinely available laboratory parameters. In normal-weight individuals, a modest elevation of WBC count above the observed cut-off (approximately 7.1 × 10^3^/µL) may serve as an inexpensive signal to prompt further metabolic assessment for example, fasting glucose and insulin for HOMA-IR estimation, lipid profiling, and measurement of waist circumference rather than as a diagnostic test in itself. In overweight individuals approaching the obesity threshold, an elevated CRP/HDL ratio (above approximately 0.045 in our cohort) appears to capture additional risk beyond BMI alone and may similarly be used to trigger targeted metabolic evaluation and earlier lifestyle counselling. Positioned within the broader literature, which shows that metabolically unhealthy normal-weight and mildly overweight individuals are common and carry substantial cardiometabolic risk that is often missed by BMI alone (Morino, Petersen & Shulman, 2006; Ruderman et al., 1998; Stefan, Schick & Häring, 2017), this framework offers a low-cost, widely accessible complement to existing risk-stratification strategies. The intended use is not diagnostic; rather, it is to refine early identification of non-obese adults who might benefit from intensified lifestyle intervention before overt metabolic disease develops. Prospective studies are required to test whether such BMI-tailored inflammatory triage can improve clinically meaningful outcomes in the non-obese population.

Several limitations of this study should be acknowledged. The retrospective and cross-sectional design limits the ability to establish causality, and the single-centre setting may restrict generalisability. Insulin resistance was defined using a single HOMA-IR cut-off value, which may not fully capture age, sex, or ethnicity-specific variability; this fixed threshold may have contributed to misclassification, particularly in younger individuals and women. Although the waist-to-hip ratio correlates more closely with insulin resistance, BMI was retained as the primary stratifier to ensure direct comparability with WHO-based literature and routine clinical use. Metabolic syndrome, potentially present despite non-obese BMI, was not formally assessed due to the lack of recordings and therefore could not be adjusted for. Owing to the limited number of insulin-resistant cases, the number of variables included in multivariable models was restricted to reduce the risk of overfitting. Lifestyle factors known to influence insulin sensitivity and metabolic risk, including smoking status and alcohol consumption, were not recorded and therefore could not be incorporated into the analyses. In addition, systematic data on inherited or monogenic conditions known to affect insulin sensitivity such as familial partial lipodystrophy or maturity-onset diabetes of the young, were not available in the retrospective database, although patients with such established diagnoses would have been excluded through the broader exclusion criteria. A further methodological limitation is that inflammation was quantified using standard C-reactive protein rather than high-sensitivity CRP, which is increasingly used in cardiometabolic research; because this was a retrospective study relying on the laboratory variables already recorded at the time of outpatient admission, remeasurement with a high-sensitivity assay was not feasible. The absence of an *a priori* sample size calculation and the relatively small overweight subgroup may have reduced statistical power and contributed to wide confidence intervals.

## Conclusions

In conclusion, low-grade systemic inflammation is closely associated with insulin resistance even in the absence of obesity. Routine laboratory parameters, particularly WBC count, may provide insight into early metabolic risk in non-obese individuals. Notably, the predictive performance of inflammatory markers differed according to body mass index, with WBC count showing greater discriminative ability in normal-weight individuals and the CRP/HDL ratio emerging as a more informative marker in overweight subjects. These findings support a BMI-tailored interpretation of inflammatory markers for metabolic risk stratification in non-obese populations.

##  Supplemental Information

10.7717/peerj.21769/supp-1 Supplemental Information 1 SPSS version of raw data

10.7717/peerj.21769/supp-2 Supplemental Information 2 Participant-level SPSS (.sav) dataset supporting the cross-sectional analysis of CRP/HDL, NLR, SII, and PLT/MPV ratios in relation to BMI category and HOMA-IR–defined insulin resistance. Baseline demographic, anthropometric, and biochemical characteristics of the study population stratified by insulin resistance status (n = 149). Participants were classified as insulin-resistant (HOMA-IR ≥ 2.5) or non-insulin-resistant (HOMA-IR < 2.5). Continuous variables are presented as [mean ± SD or median (IQR), as appropriate]; categorical variables are presented as *n* (%). Between-group comparisons were performed using [Student’s *t*-test/Mann–Whitney *U* test] for continuous variables and [chi-square/Fisher’s exact test] for categorical variables. Abbreviations: BMI, body mass index; CRP, C-reactive protein; HDL, high-density lipoprotein cholesterol; HOMA-IR, homeostasis model assessment of insulin resistance; IR, insulin resistance; WBC, white blood cell.

10.7717/peerj.21769/supp-3 Supplemental Information 3 Categorical Data listed in the raw file

10.7717/peerj.21769/supp-4 Supplemental Information 4 STROBE Checklist

## Additional Information and Declarations

### Data Availability

The following information was supplied regarding data availability: The raw measurements are available in the Supplemental Files.

### Clinical Trial Registration

The following information was supplied regarding Clinical Trial registration: Decision No: 2025/13-16; Date: November 18, 2025
