# Supplementary material for: Body mass index–stratified inflammatory predictors of insulin resistance in non-obese adults: a retrospective study
Source: PeerJ. 2026 Oct 2;14:e21769. doi: 10.7717/peerj.21769 (PMC13637635; doi:10.7717/peerj.21769)
Supplement: Supplementary file 1 [file peerj-14-21769-s003.docx]

Categorical Data

Name: Gender

Explanation: 0: Man, 1: Woman

Name: BMI_Normal_Owerweight

Explanation: 0: Normal weight (BMI<25), 1:BMI<30

Name: IR_1_0_binary

Explanation: 0: Insulin Resistance (-), 1: Insulin Resistance (+)
